# T Cells Infiltrating Diseased Liver Express Ligands for the NKG2D Stress Surveillance System

**DOI:** 10.4049/jimmunol.1601313

**Published:** 2016-12-28

**Authors:** Wei-Chen Huang, Nicholas J. Easom, Xin-Zi Tang, Upkar S. Gill, Harsimran Singh, Francis Robertson, Chiwen Chang, John Trowsdale, Brian R. Davidson, William M. Rosenberg, Giuseppe Fusai, Antoine Toubert, Patrick T. Kennedy, Dimitra Peppa, Mala K. Maini

**Affiliations:** * Division of Infection and Immunity, University College London, London WC1E 6JF, United Kingdom;; † Tri-Service General Hospital, National Defense Medical Center, Taipei 114, Taiwan;; ‡ Centre for Immunobiology, Blizard Institute, Bart’s and the London School of Medicine and Dentistry, Queen Mary University of London, London E1 2AT, United Kingdom;; § Institute for Liver and Digestive Health, University College London, London NW3 2PF, United Kingdom;; ¶ Department of Surgery and Interventional Science, University College London, London WC1E 6BT, United Kingdom;; ‖ Department of Pathology, University of Cambridge, Cambridge CB2 1QP, United Kingdom; and; # Université Paris Diderot, Sorbonne Paris Cité, INSERM UMRS 1160, AP-HP, Hôpital Saint-Louis, Paris 75013, France

## Abstract

NK cells, which are highly enriched in the liver, are potent regulators of antiviral T cells and immunopathology in persistent viral infection. We investigated the role of the NKG2D axis in T cell/NK cell interactions in hepatitis B. Activated and hepatitis B virus (HBV)–specific T cells, particularly the CD4 fraction, expressed NKG2D ligands (NKG2DL), which were not found on T cells from healthy controls (*p* < 0.001). NKG2DL-expressing T cells were strikingly enriched within HBV-infected livers compared with the periphery or to healthy livers (*p* < 0.001). NKG2D^+^NK cells were also increased and preferentially activated in the HBV-infected liver (*p* < 0.001), in direct proportion to the percentage of MICA/B-expressing CD4 T cells colocated within freshly isolated liver tissue (*p* < 0.001). This suggests that NKG2DL induced on T cells within a diseased organ can calibrate NKG2D-dependent activation of local NK cells; furthermore, NKG2D blockade could rescue HBV-specific and MICA/B-expressing T cells from HBV-infected livers. To our knowledge, this is the first ex vivo demonstration that non-virally infected human T cells can express NKG2DL, with implications for stress surveillance by the large number of NKG2D-expressing NK cells sequestered in the liver.

## Introduction

Natural killer cells are well known for their capacity to kill virally infected and transformed cells, but also have potent regulatory capacity (1–3). In particular, their ability to modulate antiviral T cell responses, thereby regulating immunity and immunopathology, has been highlighted by several studies in murine CMV and lymphocytic choriomeningitis virus (4–9). We demonstrated the relevance of this in humans with persistent hepatitis B virus (HBV) infection, where NK cells were able to delete HBV-specific CD8 T cells in a rapid, contact-dependent manner (10). More recently, HBV-specific CD4 T cells have also been shown to be susceptible to NK regulation in patients with HBV suppressed by antivirals (11). We postulated that interactions between NK cells and T cells would be accentuated in the HBV-infected liver, where NK cell frequencies are greatly enriched, T cells are dysregulated, and cell to cell contact is facilitated by the narrow-lumen, low flow rate of the liver sinusoidal vasculature.

NK cells are the most prevalent lymphocyte population in the human liver, accounting for up to a third of intrahepatic leukocytes (12, 13). In addition to conventional bone marrow–derived NK cells, recent studies in mice have defined a specialized hepatic-specific lineage of NK cells (14–17), underscoring their relevance in the liver. Similarly, we have recently described a large subset of CXCR6^+^Tbet^lo^Eomes^hi^ NK cells residing in human liver that are not present in the circulation (18). In viral hepatitis, NK cells (particularly the liver-resident subset) upregulate TRAIL, barely expressed on healthy hepatic NK cells in humans, and can kill HBV/hepatitis C virus–infected hepatocytes bearing TRAIL death receptors (18–21). The TRAIL pathway also contributes to the capacity of NK cells to preferentially kill T cells directed against HBV and those activated within the HBV-infected liver, which we found express the death-inducing receptor TRAIL-R2 not normally found on T cells (10). Although we identified TRAIL as one effector pathway used by NK cells to delete T cells, the interactions initiating NK killing of T cells have not been elucidated in humans. We hypothesized that T cells in the metabolically stressed environment of the HBV-infected liver may upregulate specific ligands to activate NK cell cytotoxicity.

We focused on the NKG2D axis because this plays a critical role in lymphoid stress surveillance within tissues (22–24). The relevance of this pathway was supported by the fact that NK cells maintain high levels of the major activatory receptor NKG2D in HBV (25, 26), and NKG2D-dependent killing of T cells has been demonstrated in vitro (27–31) and in murine models in vivo (4). Various stressors (oxidative, genotoxic, viral infection) can induce epithelial cells to express one or more ligands for NKG2D, as a delicately balanced system for regulating immunopathology (22, 32). Likewise, human T cells have been shown to have inducible expression of NKG2D ligands (NKG2DL) when exposed to mitogens or other stimuli and/or infected with CMV or HIV in vitro (28, 33–38). To our knowledge, in this study we demonstrate for the first time the in vivo induction of NKG2DL on uninfected human T cells, particularly on activated and virus-specific CD4 T cells within the HBV-infected liver milieu. We provide ex vivo data suggesting that CD4 T cell expression of NKG2DL can drive local NK cell activation in a dose-dependent manner.

## Materials and Methods

### Patients and healthy controls

Blood samples were obtained from 113 chronic hepatitis B (CHB) patients recruited from the Mortimer Market Clinic (Central and North West London National Health Service Trust), the Royal Free and University College London hospitals and the Royal London Hospital (Barts Health National Health Service Trust). A total of 46 healthy control blood samples were obtained from staff or students at University College London. Surplus liver tissue was obtained from 36 CHB patients undergoing diagnostic liver biopsies. Healthy liver remote from the tumor site was obtained from 11 non-HBV infected patients undergoing tumor resection for colorectal metastases. Nine transplant perfusates from cadaveric donor livers were collected during liver transplant surgery under the standard graft preparation protocols. The study was approved by the relevant ethical review boards and informed consent was obtained in writing. All CHB patients had detectable hepatitis B surface Ag and DNA for >6 mo, were anti-hepatitis C virus, anti-δ virus and anti-HIV–Ab negative and treatment naive. Liver inflammation was determined by serum alanine transaminase (ALT). Details of the clinical characteristics of all HBV patients are included in Table I.

### PBMC and intrahepatic lymphocyte isolation

PBMC isolated from whole blood by standard gradient centrifugation on Ficoll-Paque Plus (GE Healthcare). Intrahepatic lymphocytes (IHL) were obtained following gentle mechanical disruption of liver tissue prior to passing through a 70 μm cell strainer (BD Biosciences) and multiple washes with RPMI 1640 (Invitrogen) as previously described (13, 20). IHLs from transplant perfusates were retrieved by density-gradient centrifugation.

### Flow cytometric analysis and Abs

For phenotypic analysis, PBMC and IHL were washed with PBS and stained with a fixable Live/Dead blue dye (Invitrogen) at 4°C for 10 min, prior to Fc receptor blocking with FcR blocking reagent (Miltenyi Biotec, Bergisch Gladbach, Germany). Surface staining was performed at 4°C for 30 min in the presence of saturating concentrations of mAbs, or isotype matched controls. The following Abs were used for the sequential gating strategy (Supplemental Fig. 1A): CD3-PE-Cy7 (eBioscience), CD4-APC-eFluor780 (eBioscience), CD8-AlexaFluor700 (eBioscience), CD19-APC-eFluor780 (eBioscience), CD56-ECD (Beckman Coulter), HLA-DR-V500 (BD Biosciences), NKG2D-Alexa Fluor 488 (R&D Systems), MICA/B-PE (eBioscience), ULBP1-FITC (R&D Systems), ULBP2/5/6-APC (R&D Systems), and ULBP3-PE (R&D Systems). The frequencies of peptide-specific CD8 T cells from HLA-A2 positive individuals were evaluated directly ex vivo by multimer staining as previously described (39). Briefly, total PBMCs were stained with APC-labeled CMV pp65 495–504 or HBV core 18–27, envelope 183–191, envelope 335–343, envelope 348–357, and polymerase 508–510 dextramers (Immudex, Denmark) at 37°C for 15 min in complete RPMI 1640 plus 10% FCS. The cells were then washed, pelleted, and stained as above. A control dextramer was used to identify the population of positive cells. Intracellular staining (ICS) to assess proliferation was performed using Ki67-FITC (BD Biosciences) mAb directly ex vivo. ICS for IFN-γ or active caspase 3 levels was carried out by using IFN-γ V450, (BD Biosciences) or Caspase 3-PE (BD Biosciences) mAbs in 0.1% saponin for 30 min. All samples were acquired on a LSR Fortessa or BD LSR II using BD FACSDiva 6.0 (BD Biosciences). Data were analyzed using FlowJo v.8 (TreeStar, Ashland, OR).

### Cell lines

A 2B4 cell line carrying GFP–conjugated NF-AT transfected with the human NKG2D/DAP10-CD3ζ complex as previously described (40) was used to screen for NKG2DL on PBMC. B-lymphoblastoid cell lines, C1R and C1R transfected with MICA*008 (C1R-MICA) were used as a source of cell-bound MICA, expressing 0 and 100% cell-bound MICA respectively; anti-CD19 APC-eFluor 780 (eBioscience) was used to identify the cells and MICA expression was verified by MICA/B mAb staining as previously described (41).

### NK and CD4 T cell isolation

NK cells and CD4 T cells were isolated from PBMC (>95% purity and viability) (NK isolation kit and CD4 T cell isolation kit respectively; Miltenyi Biotec, Bergisch Gladbach, Germany) according to manufacturer’s instructions.

### NKG2DL expression and in vitro stimulation

The presence of NKG2DL on PBMC or isolated CD4 T cells from CHB patients was investigated in cocultures with GFP reporter cells. Cells were cocultured with 2B4 transfectants overnight in RPMI 1640 containing 5% FCS, and GFP expression analyzed by flow cytometry. Purified CD4 T cells or PBMC from healthy controls were rested for 48 h prior to the addition of H_2_O_2_ solution (30%; BDH Aristar) at varying concentrations (0.25–1 mM) for 0.5–2 h, followed by surface staining for NKG2DL as described above.

### NK cell degranulation

To measure NK cell capacity to degranulate, purified NK cells were cocultured at 1:1 ratio with target cells (C1R/C1R-MICA) at 37°C for 6 h, in the presence of anti-CD107a-APC (BD Biosciences). Brefeldin A (1 μg/ml; Sigma-Aldrich) and Monensin (1 μg/ml; Sigma-Aldrich) were added after 1 h. Where indicated, an anti-NKG2D blocking Ab or control Ab mAb (0.5 μg/ml; eBiosciences) was added at the onset of culture. NKG2D blockade did not affect NK cell viability.

### Short-term culture and NKG2D blockade

PBMC were stimulated with HBV overlapping peptides (OLP) (15mer peptides overlapping by 10 residues spanning genotype D HBV core protein, 1 μg/ml; JPT) in the presence of 40 IU IL-2 (Miltenyi Biotec) in RPMI 1640 complete medium for 10 d at 37°C. IL-2 and medium were refreshed on day 4 of culture. On day 9, PBMC were restimulated with 1 μg/ml OLP overnight, in the presence of Brefeldin A (1 μg/ml). Virus-specific T cells were identified via ICS for corresponding IFN-γ production. To examine the effect of blocking NKG2D on virus specific CD4 T cells, NKG2D blocking (0.5 μg/ml, eBiosciences) or an isotype-matched control mAb were added with peptide at onset of culture and cells were treated as described above.

### Overnight stimulation

For overnight stimulation of PBMC or IHL, 10 μg/ml HBV OLPs were added and the cells were incubated at 37°C in the presence of Brefeldin A (1 μg/ml) (added after 1 h). HBV-specific T cells were identified by ICS for IFN-γ.

### Statistical analysis

The non-parametric Mann–Whitney *U* test (for two groups), the Wilcoxon signed rank test (for two paired groups), Kruskal–Wallis (for >2 non-paired groups) or Friedman (for >2 paired groups) one-way tests were used as appropriate. Correlations between variables were analyzed using Spearman’s rank correlation coefficient (*r*). A *p* value < 0.05 was considered significant for all tests. All figures are marked as follows: *p < 0.05, **p < 0.005, ***p < 0.001.

## Results

### Induction of NKG2DL on T cells in CHB

**FIGURE 1.**
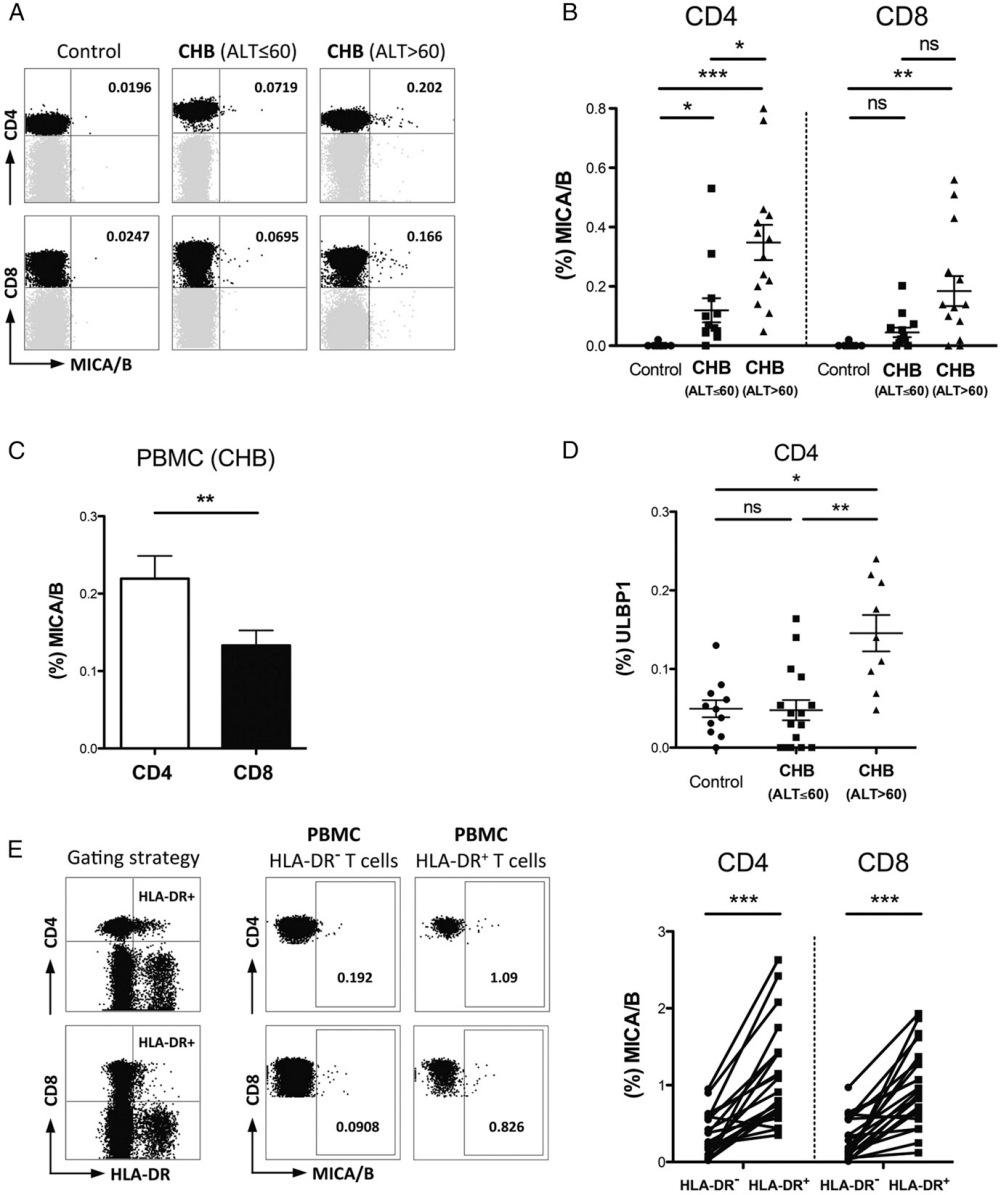
NKG2DL induction on activated T cells in patients with CHB. Representative FACS plots gated on live CD3^+^ (**A**) and summary data (**B**) of ex vivo MICA/B expression on global CD4 and CD8 T cells from healthy controls (*n* = 9), CHB patients with ALT ≤60 IU/l (*n* = 13) or ALT >60 IU/l (*n* = 14), analyzed by Kruskal–Wallis test (gates set using isotype controls). (**C**) Comparison of MICA/B expression on peripheral CD4 and CD8 T cells from CHB patients (*n* = 27), analyzed by Wilcoxon signed rank test. (**D**) Summary results of ex vivo ULBP1 mAb staining of global CD4 T cells from healthy controls (*n* = 11), CHB patients with ALT ≤60 IU/l (*n* = 16) or ALT >60 IU/l (*n* = 9), analyzed by Kruskal–Wallis test. (**E**) Representative FACS plots (gated on live CD3^+^) and summary data of MICA/B staining of HLA-DR^−^ and HLA-DR^+^ CD4 and CD8 T cells from the periphery (*n* = 22) of patients with CHB, analyzed by Wilcoxon signed rank test. *p < 0.05, **p < 0.005, ***p < 0.001.

The expression of NKG2DL is low in healthy tissues, but can be induced by cellular stress such as infection, inflammation, or tumor transformation (22, 32). We hypothesized that NKG2DL may be upregulated on T cells in CHB, allowing them to interact with NKG2D^+^NK cells in the HBV-infected liver. To investigate NKG2DL expression, CD4 and CD8 T cells from healthy controls and a well-characterized cohort of patients with CHB (Table I) were evaluated by flow cytometry (Supplemental Fig. 1A) using a panel of mAbs specific for MICA/B, ULBP1, ULBP2/5/6, and ULBP3. Out of this panel of ligands, MICA/B was not detectable on T cells from controls but showed subtle but significant induction on peripheral CD4 and CD8 T cells in patients with CHB; T cell MICA/B levels did not correlate with virological parameters but were more increased in those with more pronounced liver inflammation (serum ALT >60 IU/l, Fig. 1A, 1B). MICA/B was preferentially expressed on CD4 compared with CD8 T cells (Fig. 1C). ULBP1 was also selectively elevated on CD4 T cells from patients with HBV-related liver inflammation (serum ALT >60 IU/l, Fig. 1D). However, no significant changes were observed for the T cell expression of the remaining NKG2DL (ULBP2/5/6 and ULBP3) in CHB (data not shown). As a further readout, we screened with a reporter cell line expressing NKG2D tagged to GFP and able to bind all NKG2DL, again confirming that their levels were selectively enriched in patients with HBV-related liver inflammation (Supplemental Fig. 1B).

**Table I.** Clinical data of study controls and patients

	Healthy Controls (*n* = 46) (PBMC)	CHB (*n* = 69) (PBMC, ALT ≤60)	CHB (*n* = 44) (PBMC, ALT >60)	CHB Liver (*n* = 36) (Biopsy)	Control Liver (*n* = 9) (Perfusate)
Age, y (mean ± SEM)	32.4 ± 1.4	37.8 ± 1.3	37.1 ± 1.8	37.8 ± 1.7	42.2 ± 6.2
Gender, female/male (%)	56/44	36/64	52/48	33/67	44/56
ALT IU/l [median (interquartile range)]	n.a.	30.1 (21–37)	142.4 (80–168)	32.5 (24.3–76.8)	n.a.
HBeAg, positive/negative (%)	n.a.	17.4/82.6	56.8/43.2	25/75	n.a.
HBV DNA copies/ml [median (range)]	n.a.	1.4 × 10^3^ (20–3.2 × 10^8^)	4.1 × 10^6^ (860–1.1 × 10^9^)	4.5 × 10^3^ (20–6.5 × 10^5^)	n.a.

n.a., not applicable

Although NKG2DL were consistently increased on T cells from patients with CHB, the percentage of expressing cells in the peripheral circulation was low, suggesting that a subset of T cells was selectively affected. We hypothesized that NKG2DL might be preferentially induced on those T cells activated by the HBV-infected liver milieu. Using direct ex vivo staining, we found an enrichment of MICA/B expression on the circulating, activated (HLA-DR^+^) fraction compared with HLA-DR^−^ T cells, particularly in the CD4 compartment (Fig. 1E).

Previous reports have shown upregulation of NKG2DL following in vitro induction of T cell proliferation (28). However, a simple relationship between antigenic proliferation and NKG2DL induction was not supported by our findings; there was no ex vivo correlation with viral load and no increase in proliferating (Ki67^+^) T cells within the NKG2DL-expressing fraction (Supplemental Fig. 1C). Reactive oxygen species, leading to oxidative stress, have also been shown to induce NKG2DL on cell lines (28, 42) and DNAM-1 ligand on T cells (43), and are known to be elevated in the liver in HBV-related pathology (44, 45). A possible role for oxidative stress in CHB was supported by the fact that in vitro exposure to H_2_O_2_ recapitulated the pattern of induction of NKG2DL seen on T cells from patients. Following 1 h exposure to H_2_O_2,_ MICA/B was consistently induced on purified CD4 T cells from 14 healthy controls (Supplemental Fig. 1D) and to a lesser extent on CD4 T cells within PBMC (Supplemental Fig. 1E). The H_2_O_2_-induced upregulation of NKG2DL on CD4 within PBMC was most striking for MICA/B and ULBP-1 (Supplemental Fig. 1E, 1F), mimicking the preferential increase of these ligands in our ex vivo studies of CHB patients. By contrast, in vitro exposure to a large variety of other factors relevant to the liver milieu (TNF-α, IFN-γ, IFN-α, IL-8, IL-2, IL-15, IL-17, IL-10, TGF-β, EGF, l-arginine depleted medium, hypoxic incubation, LPS, and HBV viral Ags) failed to reproducibly induce NKG2DL on T cells (data not shown).

### T cells infiltrating HBV-infected livers are enriched for NKG2DL

**FIGURE 2.**
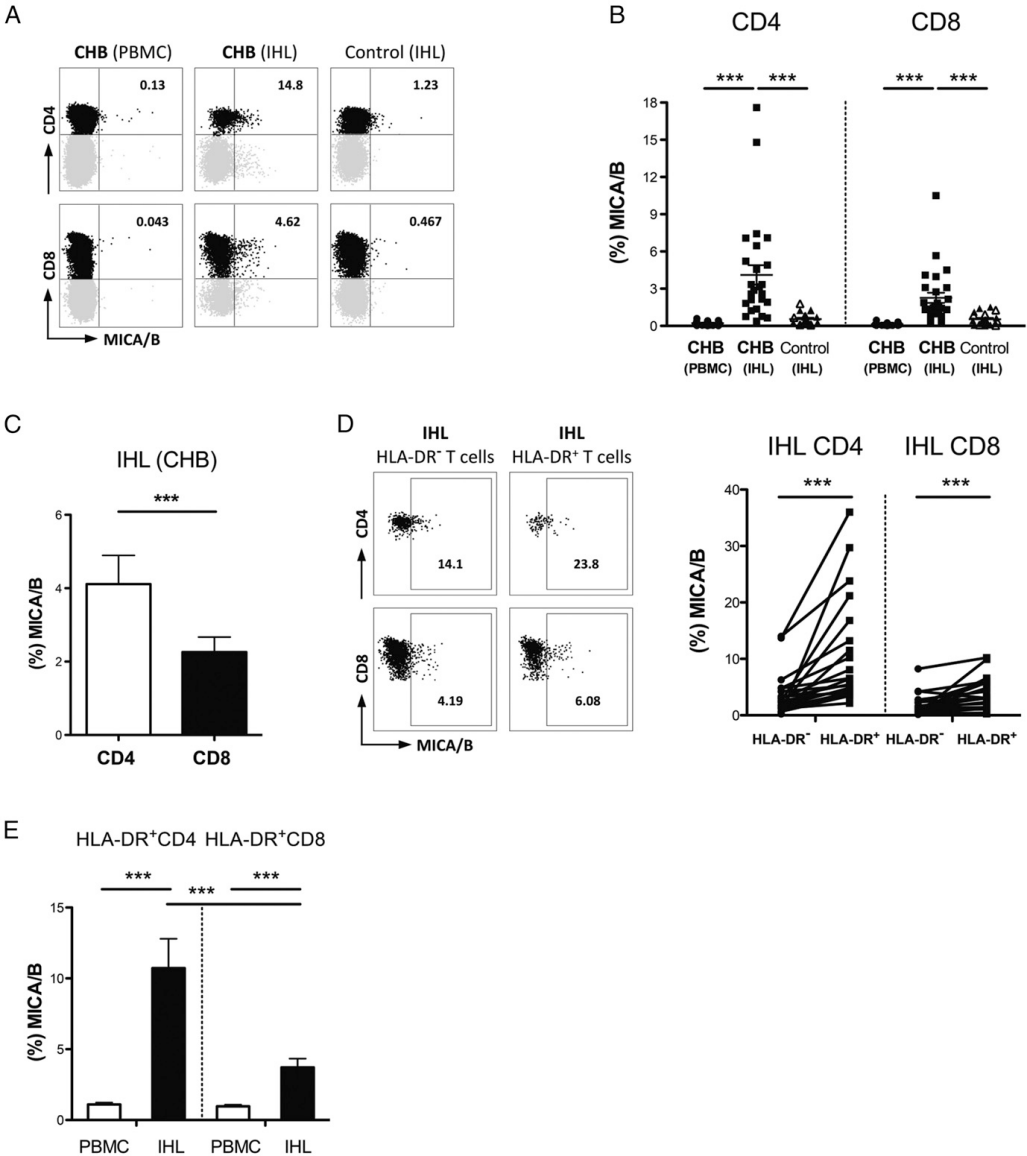
Enrichment of NKG2DL-expressing T cells within HBV-infected but not healthy liver. Representative FACS plots gated on live CD3^+^ (**A**) and summary (**B**) of ex vivo MICA/B expression on paired peripheral and intrahepatic T cells from CHB patients (*n* = 27), and intrahepatic T cells from non-HBV infected controls (intrahepatic lymphocytes isolated from healthy liver resected distant to colorectal metastases [filled triangles *n* = 11] or from transplant perfusates of deceased donor livers [open triangles *n* = 9]; analyzed by Kruskal–Wallis test). (**C**) Comparison of MICA/B expression on intrahepatic CD4 and CD8 T cells from CHB patients (*n* = 27), analyzed by Wilcoxon signed rank test. (**D**) Representative FACS plots (gated on live CD3^+^) and summary data of MICA/B staining of HLA-DR^−^ and HLA-DR^+^ CD4 and CD8 T cells from the liver (IHL *n* = 21) of patients with CHB, analyzed by Wilcoxon signed rank test. (**E**) Comparison of ex vivo MICA/B expression on HLA-DR^+^ CD4 and CD8 T cells from the PBMC (*n* = 22) and liver (*n* = 21) of CHB patients, analyzed by Mann–Whitney *U* test (comparison between CD4 and CD8 T cells in same donors by Wilcoxon signed rank test). ***p < 0.001.

To probe T cell NKG2DL expression at the site of disease pathogenesis, we isolated IHL from surplus liver biopsy tissue from 21 patients with CHB and compared these with their paired PBMC samples. IHL from 20 non-HBV infected control livers were used for comparison (isolated from either healthy livers, obtained during resection of colorectal metastases, or from transplant perfusates from cadaveric donor livers). We observed a striking increase in the proportion of T cells expressing MICA/B directly ex vivo from the liver compared with the periphery of patients with CHB. By contrast, intrahepatic T cells from control livers had low expression of MICA/B (Fig. 2A, 2B). The percentage of intrahepatic CD4 T cells expressing MICA/B in HBV-infected livers could be as high as 18%, a more than 10-fold increase of the maximum seen on circulating CD4 in CHB, or on intrahepatic CD4 of healthy livers (Fig. 2A, 2B). In the liver of patients with CHB, as in their circulation, MICA/B was preferentially expressed on CD4 compared with CD8 T cells (Fig. 2C) and on the activated fraction (HLA-DR^+^ versus HLA-DR^−^ CD4 and CD8, Fig. 2D). Within the activated (HLA-DR^+^) fraction, MICA/B was expressed on more CD4 than CD8 T cells and on more intrahepatic than peripheral T cells (Fig. 2E), consistent with the concept that factors in the HBV-infected liver environment could be driving upregulation of NKG2DL.

### Increased NKG2DL on HBV-specific T cells

**FIGURE 3.**
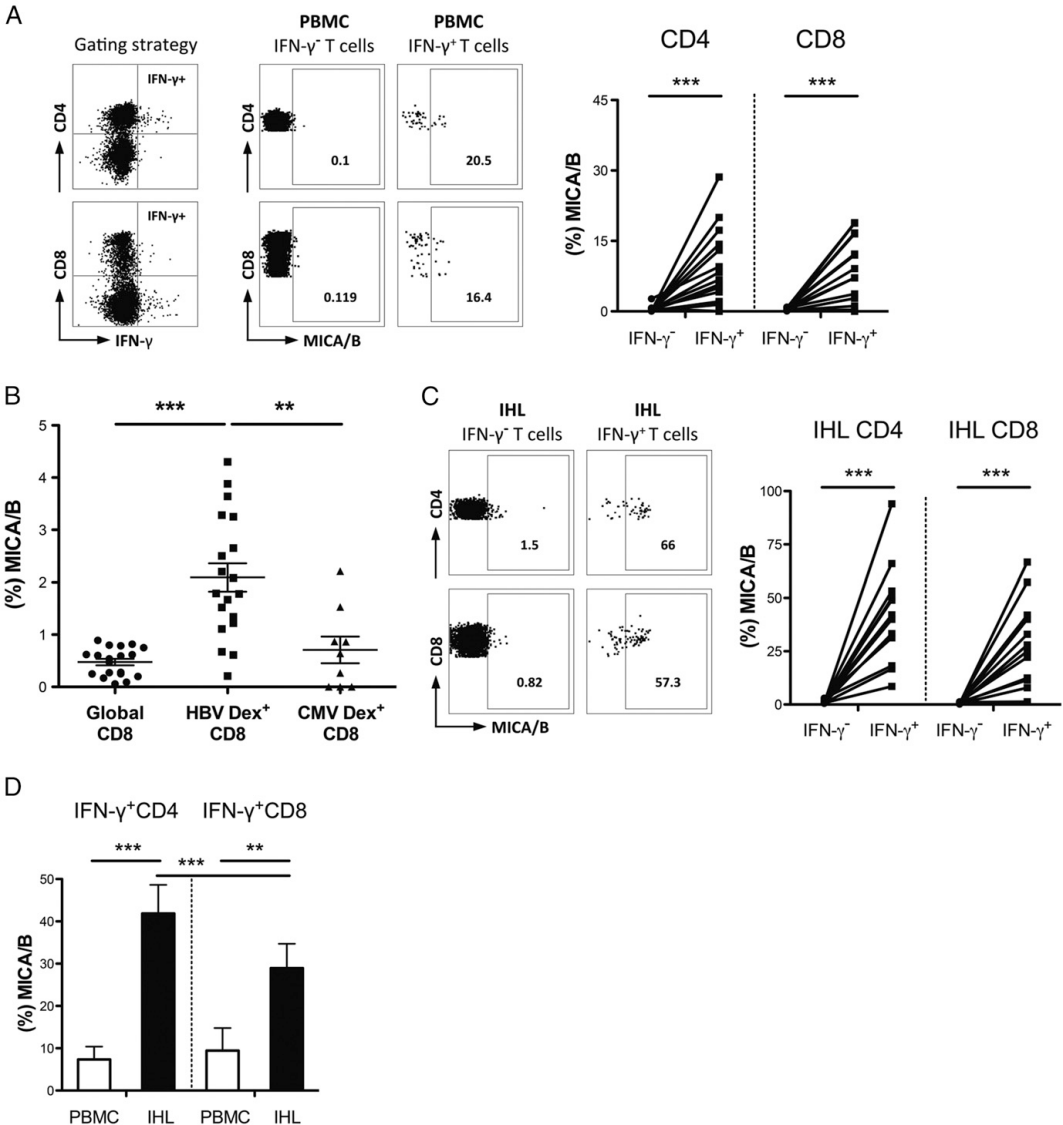
NKG2DL expression on HBV-specific T cells. Representative FACS plots (gated on live CD3^+^) and summary data of MICA/B staining of HBV-specific (IFN-γ^+^ following stimulation with OLP spanning HBV core) and IFN-γ^−^ CD4 and CD8 T cells of patients with CHB from (**A**) periphery after 10 d culture (*n* = 17) and (**C**) liver after overnight culture (*n* = 12), analyzed by Wilcoxon signed rank test. (**B**) Comparison of MICA/B expression on global, HBV/dextramer-stained and CMV/dextramer-stained ex vivo peripheral CD8 T cells from patients with CHB (*n* = 19, of which nine were CMV responders), analyzed by Kruskal–Wallis test. (**D**) Summary results of MICA/B expression on HBV-specific (IFN-γ^+^) CD4 and CD8 T cells from paired PBMC (*n* = 10) and IHL (*n* = 12 of which 10 were paired samples) following overnight HBV OLP stimulation, analyzed by Wilcoxon rank test. **p < 0.005, ***p < 0.001.

We next investigated whether HBV-specific T cells can preferentially upregulate NKG2DL. HBV-specific T cells were identified by IFN-γ production following in vitro stimulation and short-term expansion with OLP spanning the HBV core protein. After stimulation of PBMC, HBV-specific CD4 and CD8 T cells expressed MICA/B at higher levels than IFN-γ^−^ T cells (Fig. 3A). Ex vivo examination using a panel of HLA-A2/peptide dextramers (without any in vitro stimulation) likewise showed increased MICA/B on HBV-specific T cells compared with global T cells or compared with CMV-specific T cells within the same donors (Fig. 3B, Supplemental Fig. 2A).

We postulated that intrahepatic HBV-specific T cells, having recently encountered their cognate Ag within the liver-specific milieu, would be more likely to have upregulated the stress ligand MICA/B. In support of this, intrahepatic HBV-specific CD4 and CD8 T cells were considerably enriched for MICA/B expression after overnight culture with their cognate peptide (Fig. 3C). Intrahepatic T cells responding to HBV peptides showed greater induction of MICA/B than their circulating counterparts, with up to 94% of intrahepatic HBV-specific CD4 expressing MICA/B (Fig. 3C, 3D).

### HBV-specific and MICA/B-expressing T cells can be reconstituted upon NKG2D blockade

**FIGURE 4.**
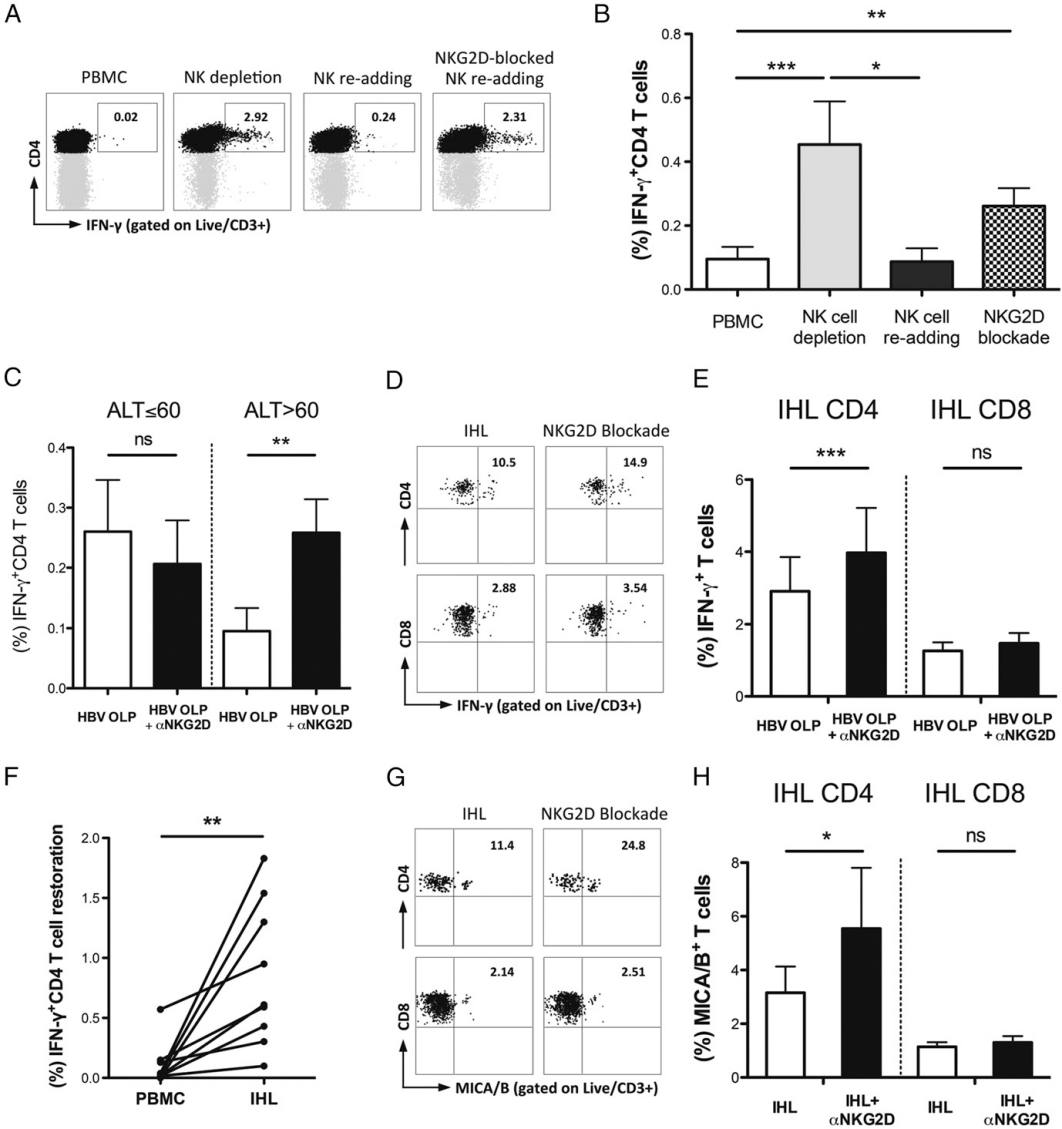
Impact of NK cells and NKG2D blockade on HBV-specific and MICA/B-expressing T cells. Representative FACS plots (**A**) of HBV-specific (IFN-γ^+^) CD4 T cell changes upon NK cell depletion, NK cell readding ^+/−^ NKG2D blockade following short-term culture of PBMC from patients with CHB with HBV OLP. (**B**) Summary data of HBV-specific (IFN-γ^+^) CD4 T cell changes upon NK-depletion, NK cell readding and NKG2D blockade following short-term culture with HBV OLP in PBMC from patients with ALT >60 IU/l (*n* = 15), analyzed by Friedman test. (**C**) Comparison of HBV-specific (IFN-γ^+^) CD4 T cell restoration upon NKG2D blockade following short-term culture with HBV OLP of PBMC from patients with ALT ≤60 IU/l (*n* = 11) and patients with ALT >60 IU/l (*n* = 15), analyzed by Wilcoxon signed rank test. Representative FACS plots (**D**) and summary data (**E**) of intrahepatic HBV-specific (IFN-γ^+^) CD4 and CD8 T cell changes upon NKG2D blockade following overnight HBV OLP stimulation in IHL from patients with CHB (*n* = 12), analyzed by Wilcoxon signed rank test. (**F**) Comparison of HBV-specific (IFN-γ^+^) CD4 T cell restoration upon NKG2D blockade following overnight HBV OLP stimulation in paired PBMC and IHL from patients with CHB (*n* = 9), analyzed by Wilcoxon signed rank test. Representative FACS plots (**G**) and summary data (**H**) of intrahepatic MICA/B expressing CD4 and CD8 T cell changes upon NKG2D blockade following overnight HBV OLP stimulation in IHL from patients with CHB (*n* = 10), analyzed by Wilcoxon signed rank test. *p < 0.05, **p < 0.005, ***p < 0.001.

To test whether the expression of NKG2DL rendered HBV-specific T cells susceptible to deletion by NK cells, we analyzed their recovery upon the removal/addition of NK cells with or without NKG2D blockade. HBV-specific CD4 T cells could be increased by NK cell depletion, eliminated by their readdition and protected by the addition of NKG2D-blocking mAbs (Fig. 4A). The capacity of HBV-specific CD4 T cells to be boosted by NK depletion or NKG2D blockade and reduced by NK cell addition is summarized for 15 patients with CHB in Fig. 4B. NKG2D-mediated reconstitution was only significant in patients with HBV-related liver inflammation (ALT >60 IU/l, Fig. 4C), in keeping with the increased NKG2DL observed we had observed in this group. NKG2D blockade was unable to mediate any additional restoration of HBV-specific CD4 T cells once PBMC were depleted of NK cells (compared with NK depletion without NKG2D blockade, Supplemental Fig. 2B), demonstrating that NK cells were required for the NKG2D-dependent killing of HBV-specific CD4 T cells.

NKG2D blockade also recovered HBV-specific CD4 but not CD8 T cells within liver-infiltrating lymphocytes (Fig. 4D, 4E), with more restoration achievable from liver lymphocytes than their circulating counterparts (Fig. 4F). In support of this deletion being partially triggered by recognition of the high levels of the NKG2DL MICA/B on HBV-specific T cells, we found that liver-infiltrating CD4 T cells expressing MICA/B^+^ were increased following NKG2D blockade (Fig. 4G, 4H).

### CD4 T cell NKG2DL induction correlates with enhanced activation of NKG2D^+^NK cells in the HBV-infected liver

**FIGURE 5.**
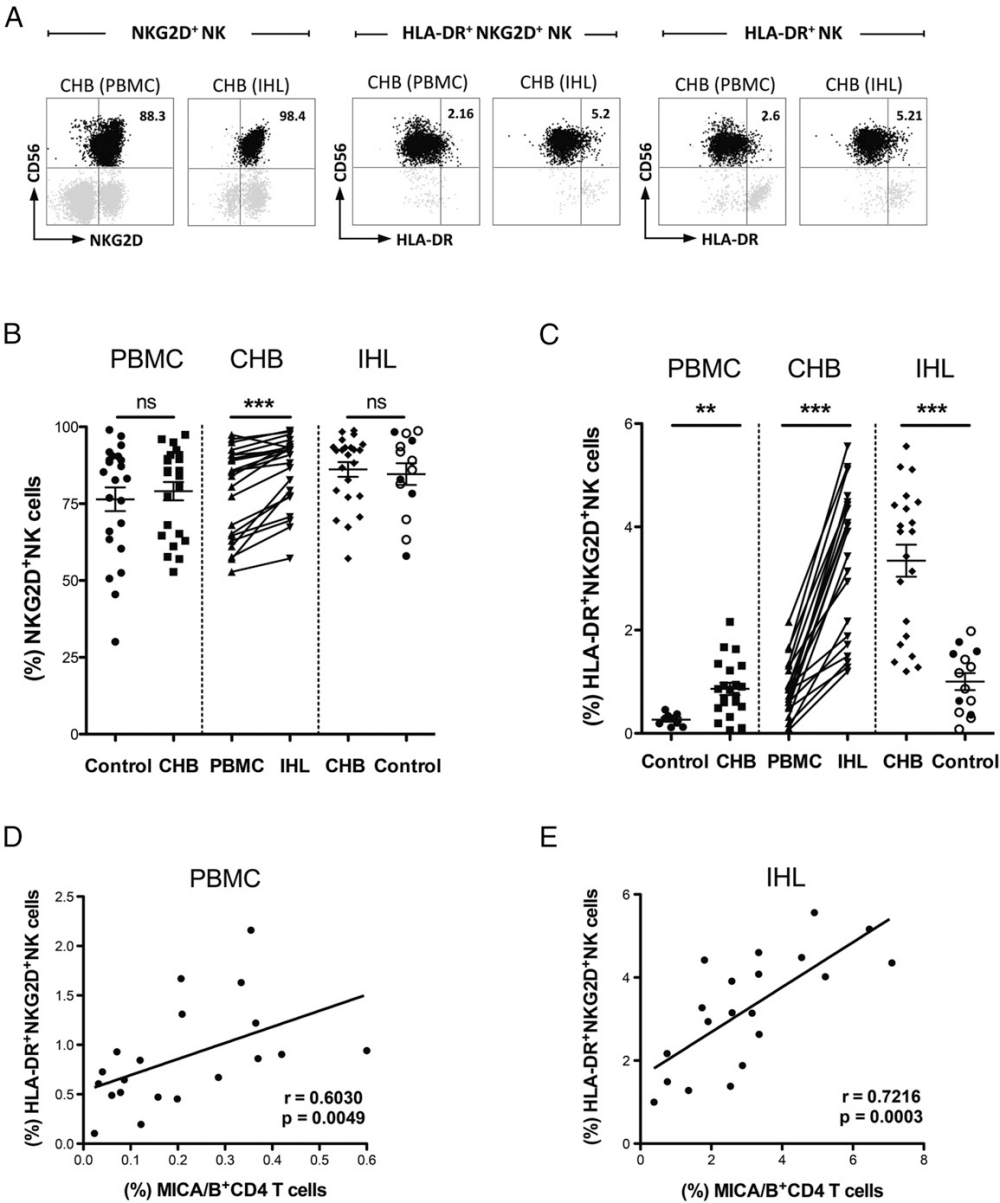
Impact of MICA-expressing cells on NK cell activation. Representative plots (**A**, gated on live CD3^−^CD56^+^) of NKG2D expression on NK cells and activation (HLA-DR) of NKG2D^+^NK/total NK cells from paired blood and liver samples from a CHB patient. Summary data (**B**) of ex vivo staining for NKG2D on NK cells of PBMC from healthy controls (*n* = 23), paired PBMC and IHL from CHB (*n* = 23), and IHL from non-HBV infected livers [filled circles (*n* = 5) IHL from healthy liver resected distant to colorectal metastases; open circles (*n* = 9) IHL from transplant perfusates of deceased donor livers], analyzed by Mann–Whitney *U* test and Wilcoxon signed rank test. Summary data (**C**) of ex vivo HLA-DR on NKG2D^+^NK cells of PBMC from healthy controls (*n* = 9), paired PBMC and IHL from CHB (*n* = 21), and IHL from non-HBV infected livers [filled circles (*n* = 5) IHL isolated from healthy liver resected distant to colorectal metastases; open circles (*n* = 9) IHL from transplant perfusates], analyzed by Mann–Whitney *U* test and Wilcoxon signed rank test. Correlation in (**D**) blood and (**E**) liver CHB samples (*n* = 20) between ex vivo MICA/B on CD4 T cells and HLA-DR on NKG2D^+^NK cells, analyzed by Spearman rank correlation test. **p < 0.005, ***p < 0.001.

To investigate the interaction of NKG2DL-expressing T cells with NK cells, we first examined if NK cells in the same HBV-infected livers maintained NKG2D expression. Although downregulation of NKG2D has been reported in some situations of pathologic overstimulation (46–48), we found no evidence for this in CHB. Both peripheral and intrahepatic NK cells from patients with CHB maintained a comparable level of NKG2D to those from non-HBV infected controls (Fig. 5A, 5B). Unexpectedly, analysis of paired samples revealed that NKG2D was even more highly expressed on intrahepatic (mean 86% NKG2D^+^NK cells and increased mean fluorescence intensity) than peripheral NK cells in CHB, and was at a similarly high level on NK cells in healthy livers (Fig. 5A, 5B, Supplemental Fig. 3A).

We then compared the activation status of peripheral and intrahepatic NKG2D IFN-γ–expressing NK cells from patients with CHB to those from healthy controls (healthy liver from resections for metastases or transplant perfusates of deceased donor livers). The NKG2D^+^ fraction of peripheral NK cells was more activated (HLA-DR^+^) in CHB than healthy donors (Fig. 5C). NKG2D^+^NK cells in HBV-infected livers showed a further highly significant increase in activation measured directly ex vivo by HLA-DR expression, compared with either their circulating counterparts or the NK cells within non–HBV-infected control livers (Fig. 5A, 5C).

To test our hypothesis that increased HLA-DR expression by NKG2D^+^NK cells in the HBV-infected liver could reflect activation by NKG2DL-expressing cells, we initially employed an in vitro system. A lymphoblastoid cell line transfected with MICA*008 (C1R-MICA, expressing 98.5% MICA^+^, Supplemental Fig. 3B) or a control cell line lacking MICA (C1R) were cocultured for 6 h with NK cells from controls or patients with CHB. MICA-expressing target cells could trigger increases in activation (HLA-DR) and degranulation (CD107) of NKG2D^+^NK cells from healthy controls and patients with CHB (Supplemental Fig. 3C), with the MICA-stimulated increase in degranulation greater for NK cells from patients with CHB (Supplemental Fig. 3D). Mixtures of the C1R-MICA and C1R-control cell line at varying ratios showed a clear dose-response of NK cells from patients with CHB to MICA-induced activation (HLA-DR) and degranulation (CD107) (Supplemental Fig. 3E, 3F). Levels of MICA expression analogous to those noted on patient T cells were able to increase the percentage of HLA-DR–expressing NK cells in the same range as observed in patient samples (Supplemental Fig. 3E).

To further probe the link between NKG2DL-expressing T cells and NK cell activation, we quantitated these two parameters in parallel using direct ex vivo analysis of blood and liver biopsy tissue from 20 patients with CHB. A highly significant correlation between the percentage of CD4 T cells expressing MICA/B and the proportion of activated (HLA-DR^+^) NKG2D^+^NK cells was seen in the periphery, and even more robustly within the liver (*r* = 0.72, *p* = 0.0003, Fig. 5D, 5E). No such correlation was found with MICA/B on CD8 T cells (data not shown).

Taken together, our analysis of tissue samples from the site of HBV pathogenesis revealed enhanced NKG2D expression and selective activation of NKG2D-expressing NK cells, in association with induction of NKG2DL on liver-infiltrating CD4 T cells. Their close ex vivo correlations suggest NKG2DL-expressing CD4 T cells may be able to calibrate NK cell activation.

## Discussion

To our knowledge, in this study we provide the first direct ex vivo evidence that uninfected human T cells are capable of expressing selective NKG2DL, and suggest that this allows them to engage in cross-talk with NKG2D-expressing NK cells. We demonstrate that the NKG2DL MICA/B is preferentially expressed on activated and HBV-specific CD4 T cells, particularly those within the HBV-infected liver. NKG2D expression is increased on NK cells from the HBV-infected liver and their enhanced activation correlates with the proportion of intrahepatic CD4 T cells expressing MICA/B. Our data reveal the novel paradigm of NKG2DL-expressing T cells sequestered in diseased livers; this raises the possibility that they will be enriched among tissue-resident populations, and contribute to organ-specific pathogenesis in other settings.

There are eight different NKG2DL in humans, which also exhibit high allelic polymorphism, providing an intricate system for signaling cellular stress and fine-tuning the response of NKG2D-bearing cells (22). We observed preferential upregulation of the ligands MICA/B and ULBP-1 on T cells from patients with CHB. By contrast, the HIV protein Vpr has been shown to selectively induce ULBP-2 on mitogen–activated HIV-infected CD4 T cells (49). The expression of particular NKG2DL on infected cells is specifically counter-regulated by a number of viral proteins in CMV or HIV infection, underscoring the importance of these signals for alerting the immune system (33, 36–38, 50). Such selective induction and repression of particular NKG2DL in response to different cellular stressors is consistent with them having evolved non-redundant roles, despite binding to a shared receptor (51). NKG2DL expression on a cell can be regulated at multiple levels, including the ATM/ATR DNA damage response driving transcription (22, 28, 50, 52) and activation of the epidermal growth factor receptor stabilizing at the posttranscriptional level (24). A further possibility not yet explored in the context of HBV infection, or liver inflammation more generally, is the shedding of NKG2DL by cleavage, exosome release, or secretion (22).

In terms of types of triggers of cellular stress, mitogen- or Ag-driven activation and proliferation have been shown to induce NKG2DL on human T cells in vitro (28), which is in line with the increase we observed on the activated and virus-specific component of the T cell pool. Our data suggested that metabolic stress within the inflamed HBV-infected liver environment could be an additional contributor to T cell NKG2DL induction. In the HBV-infected liver, T cells are highly dysregulated, and we have recently shown that they have specific metabolic defects imposed on them by arginase-producing myeloid cells (39). These, and other intrahepatic cells such as Kupffer cells, can also release reactive oxygen species (53), disrupting the local pro/antioxidant balance. Hepatic oxidative stress has been shown to drive viral hepatitis (54), including in a hepatitis B surface Ag transgenic mouse model (44). Studies in patients with CHB, in particular, staining of livers with HBV-related liver inflammation, also reveal features of oxidative stress (45, 55, 56). The possibility that T cells encountering their Ag in a milieu rich in oxidative stress are predisposed to express NKG2DL was supported by our finding that short-term in vitro exposure to hydrogen peroxide recapitulated a pattern of NKG2DL induction reminiscent of that observed in patients with CHB. This putative mechanistic link would be strengthened if future studies find that T cells from HBV-infected livers express additional markers of oxidative stress.

NKG2D-expressing NK cells can be sensitive to relatively small changes in target cell MICA/B expression (23). This was confirmed by our experiments titrating a MICA-expressing cell line and observing that as little as 10% of MICA-expressing cells could activate NKG2D-expressing NK cell cells. This frequency was modeled to be reasonably representative of the situation found within the liver sinusoids, where hepatic NK cells would first encounter HBV-specific T cells (10, 57), a large fraction of which expressed NKG2DL upon stimulation with their cognate peptide. These experiments using a class-I–deficient cell line were unable to capture the full complexity of T cell/NK cell interactions potentially coregulating NKG2D/NKG2DL effects in vivo. Nevertheless, they provided a controlled system to quantify the potential contribution of NKG2DL to NK cell activation. This was further strengthened by the ex vivo correlation observed between HLA-DR expression on NK cell and MICA/B expression on T cells, especially within the intrahepatic compartment. This suggests that stress signaling from NKG2DL-expressing intrahepatic CD4 T cells could be one factor accounting for the ∼3-fold increase in activation of NK cells in HBV-infected compared with healthy livers.

The NKG2D receptor on NK cells is primarily known for its role in the recognition of transformed and infected cells; in this study we have demonstrated its capacity to act as a sensitive detector of T cell stress in the liver. Although previous studies have shown that engagement by either cell-bound (48, 58) or soluble (47) NKG2DL can downregulate NKG2D, we found a paradoxical increase in NKG2D expression on intrahepatic NK cells. The mechanism for this counterintuitive finding is the subject of ongoing studies. Virtually all intrahepatic NK cells expressed NKG2D and were consistently more activated in the HBV-infected liver than their circulating counterparts, or than NK cells in healthy livers.

Our finding that NKG2D blockade of liver-infiltrating lymphocytes rescued HBV-specific and MICA/B-expressing CD4 but not CD8 T cells is in line with the fact that MICA/B was expressed at significantly higher levels on global, activated and HBV-specific CD4 than CD8 cells within the liver; however, it may also be attributable to CD8 T cells possessing additional mechanisms to protect them from NKG2D-mediated NK cell killing (7). Our findings are underscored by previously published work showing that NKG2D blockade rescues HBV-specific CD4 and not CD8 T cells in patients on antiviral therapy for CHB (11) and that activated NK cells from the circulation can kill T cells that have been activated in vitro to express NKG2DL (27–29). It was difficult to assess the full extent of NKG2D-dependent NK cell killing of T cells in CHB by the experimental approach we had to use, because in vitro blockade could only rescue those T cells not already deleted in vivo. The paradoxical preservation of some NKG2DL-expressing T cells in the presence of NKG2D^+^ NK cells is reminiscent of our previous finding of increased TRAIL^+^ NK cells and TRAIL-R2^+^ T cells colocated within the intrahepatic compartment (10). This could reflect the continued replacement of deleted virus-specific T cells with newly generated populations during persistent infection [as shown in lymphocytic choriomeningitis virus (59)] or the fact that a subset of T cells may be able to resist deletion, through for example NLCR5-induced MHC class I, recently described to shield T cells from NK cell mediated elimination during inflammation (60). NK cells in the HBV-infected liver also have the capacity to kill hepatocytes (20, 61), thereby contributing to liver damage; the association of T cells expressing NKG2DL with raised ALT suggests they may also promote NK cytotoxicity toward hepatocytes. Future studies should explore whether it is possible to manipulate the HBV-infected liver environment to reduce NKG2DL induction on T cells, for example with free radical scavengers, thereby potentially boosting antiviral T cells while retuning NK cell activation and cytotoxicity.

## Supplementary Material

Data Supplement
