# Supplementary material for: T Cells Infiltrating Diseased Liver Express Ligands for the NKG2D Stress Surveillance System
Source: J Immunol. 2016 Dec 28;198(3):1172–82. doi: 10.4049/jimmunol.1601313 (PMC5253436; doi:10.4049/jimmunol.1601313)
Supplement: Data Supplement [file JI_1601313.zip › JI_1601313_Supplemental_Figures_1.pdf]

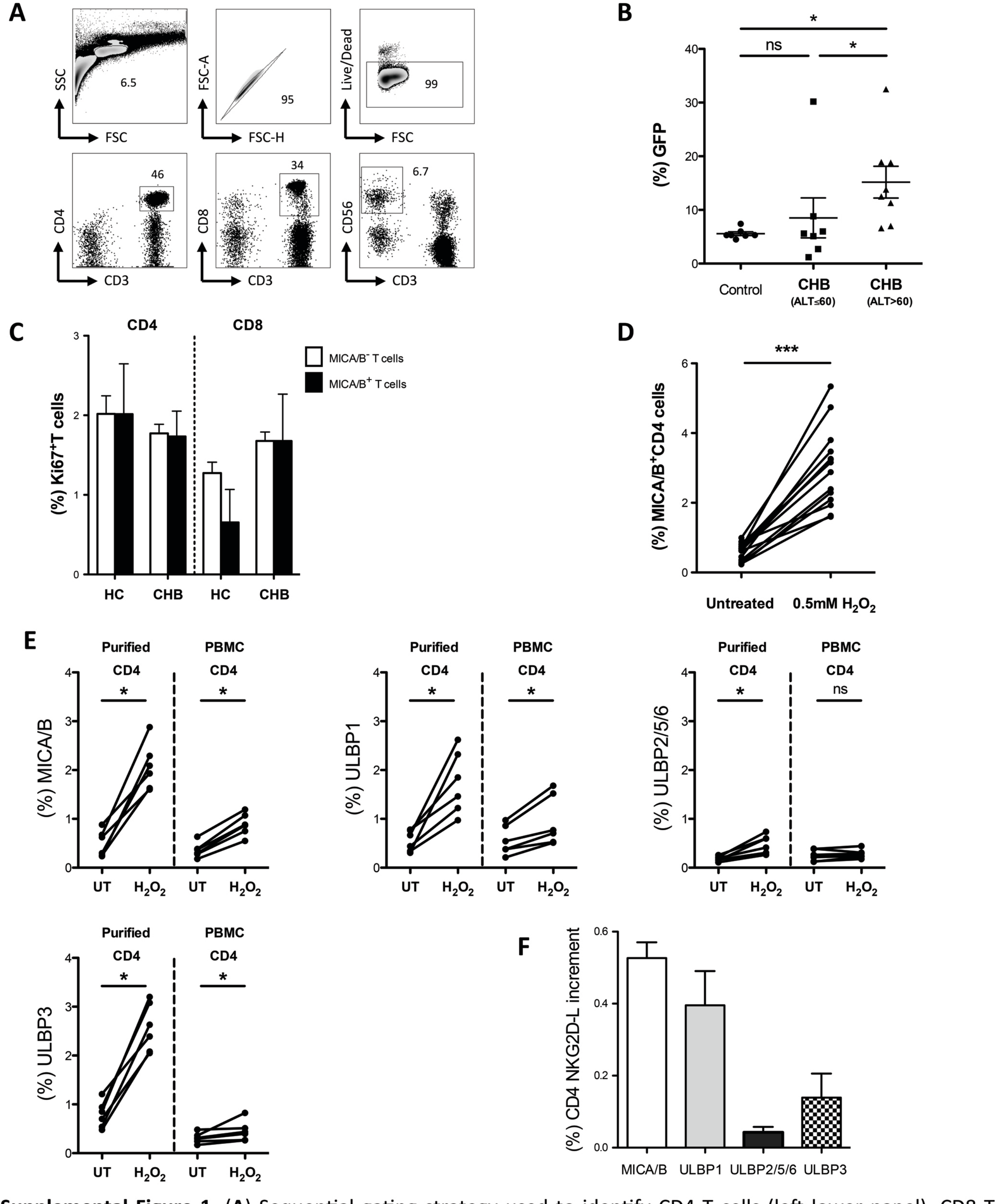

Supplemental Figure 1. (**A**) Sequential gating strategy used to identify CD4 T cells (left lower panel), CD8 T cells (middle lower panel), and NK cells (right lower panel). (**B**) Summary results of GFP reporter screening for PBMC NKG2D-L expression from healthy controls (n=7), CHB patients with ALT≤60 IU/L (n=7) or ALT>60 IU/L (n=8), analyzed by Kruskal-Wallis test. (**C**) Summary data (n=4 healthy controls, 10 CHB) of *ex vivo* Ki67 staining on MICA/B$^-$ and MICA/B$^+$ CD4 and CD8 T cells from healthy controls and patients with CHB. Induction of MICA/B expression of purified CD4 T cells from healthy donors treated with 1-hour 0.5mM H2O2 compared to untreated (UT) control cells (**D**, n=14), analyzed by Wilcoxon signed rank test. Comparison of effects of H_2O_2 treatment of purified CD4 versus whole PBMC for expression of (**E**) MICA/B, ULBP1, ULBP2/5/6, ULBP3 (n=6), analyzed by Wilcoxon signed rank test. H_2O_2-induced (treated minus untreated) pattern of NKG2D-L on CD4 T cells within PBMC of healthy controls (**F**, n=6).

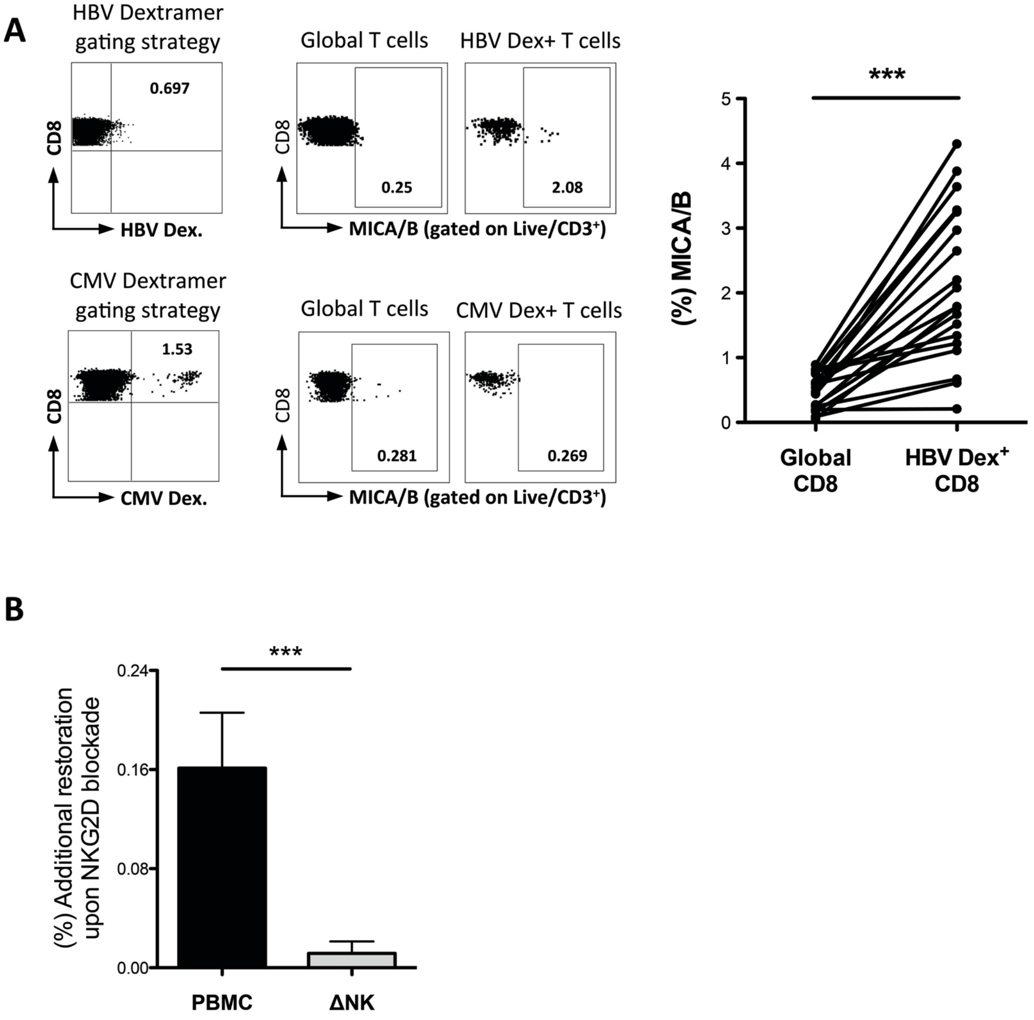

Supplemental Figure 2. (**A**) Representative plots of HBV-specific and CMV-specific (Dex$^+$) and global (Dex$^-$) peripheral CD8 T cells from patients with chronic HBV infection identified by staining with HLA-A2 restricted dextramers, and then MICA/B. Comparison of MICA/B expression on global and HBV-dextramer stained peripheral CD8 T cells (n=18), analyzed by Wilcoxon signed rank test. (**B**) Summary of HBV-specific (IFN-γ$^+$) CD4 T cell restoration upon NKG2D blockade following short-term culture with HBV OLP in PBMC with and without NK cell removal on day 0 (PBMC n=15, ΔNK n=13), analyzed by Mann Whitney test.

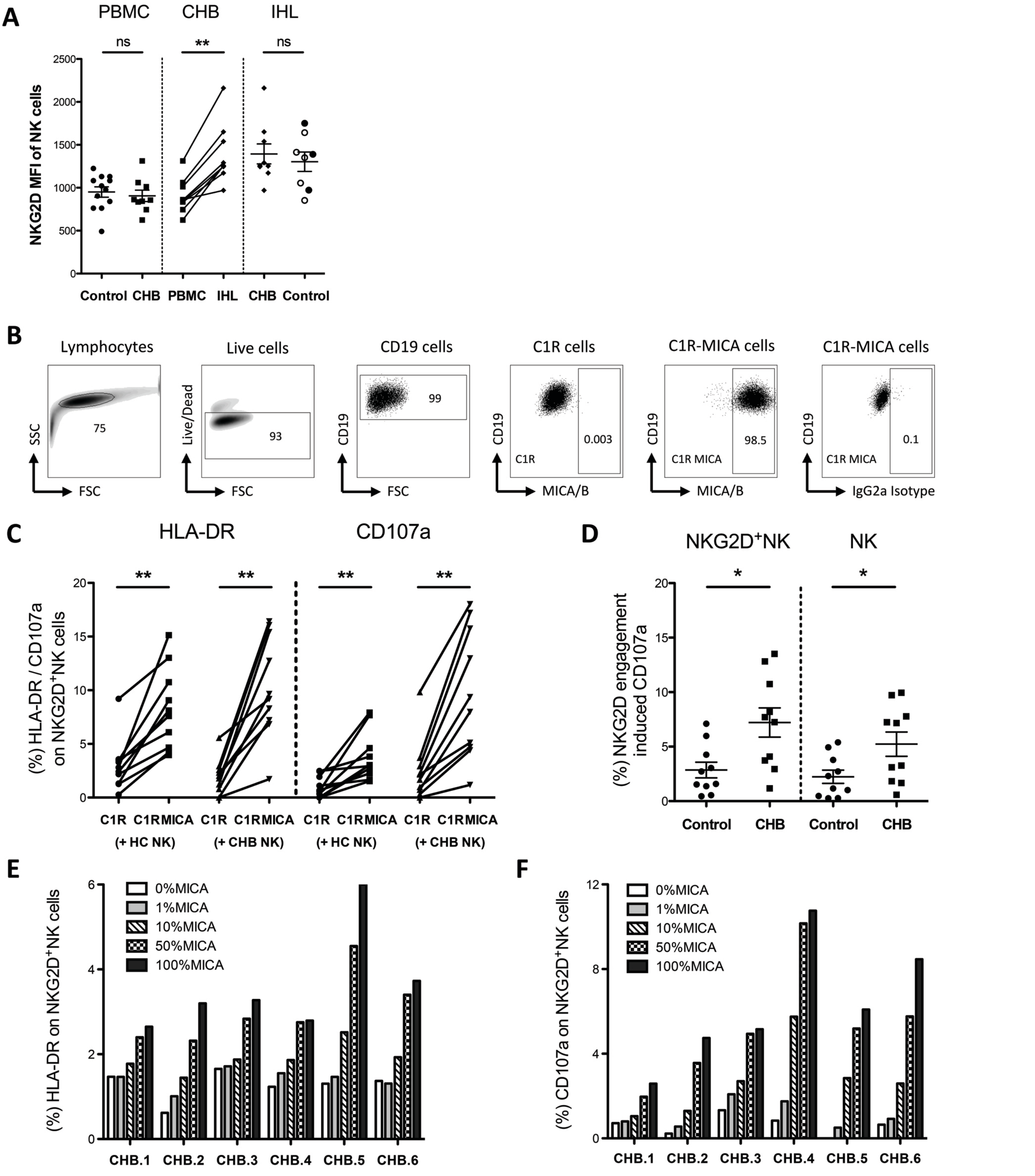

Supplemental Figure 3. (**A**) Summary data of *ex vivo* MFI for NKG2D on NK cells of PBMC from healthy controls (n=12), paired PBMC and IHL from CHB (n=9), and IHL from non-HBV infected livers (filled circles [n=3] IHL from healthy liver resected distant to colorectal metastases; open circles [n=5] IHL from transplant perfusates of deceased donor livers). (**B**) Representative plots of C1R and C1R-MICA cells showing MICA/B expression. Summary of NKG2D⁺NK cell activation and degranulation (HLA-DR/CD107a after subtraction of baseline levels) for isolated NK cells from healthy controls (n=10), CHB (n=10) co-cultured with C1R or C1R-MICA cells (**C**), analyzed by Wilcoxon signed rank test. Percentage increase in CD107a of total NK/NKG2D⁺NK cells with C1R-MICA after subtraction of C1R alone (**D**), analyzed by Mann Whitney test. Summary results of NKG2D⁺NK cell (**E**) activation (HLA-DR) and (**F**) cytotoxicity (CD107a) upon co-culture of isolated NK cells (n=6 CHB) and target cells with different MICA expression levels (after subtraction of baseline levels).
